# Applicability of creatinine-based glomerular filtration rate assessment equations to patients with neurogenic bladder

**DOI:** 10.3389/fphys.2024.1501161

**Published:** 2024-12-09

**Authors:** Panqi He, Limin Liao

**Affiliations:** ^1^ Department of Urology, School of Rehabilitation, China Rehabilitation Research Center, Beijing Bo’ai Hospital, Capital Medical University, Beijing, China; ^2^ China Rehabilitation Science Institute, Beijing, China

**Keywords:** neurogenic bladder, serum creatinine, renal function, glomerular filtration rate, ^99m^Tc-DTPA renal dynamic imaging

## Abstract

**Purpose:**

Glomerular filtration rate (GFR) measured by dynamic renal scintigraphy (Gates method) is used in this study as the standard to investigate the applicability of two creatinine (Cr)-based GFR estimation equations in Chinese patients of different genders, age groups, and GFR stages diagnosed with neurogenic bladder (NB).

**Methods:**

GFR values were measured using ^99m^Tc-DTPA renal dynamic imaging, the new serum creatinine (Cr)-based chronic kidney disease epidemiology collaborative group (CKD-EPI) equation, and the equation for the estimated GFR of CKD patients in China, which were designated as sGFR, EPI-GFR, and cGFR, respectively. Pearson’s correlation and linear regression were used to compare the differences, absolute differences, precision, and accuracies of the results of the two equations with sGFR to determine the formula offering better performance for the assessment of patients with NB.

**Results:**

Measurements from a total of 99 patients with NB were used in the final analysis. Both cGFR and EPI-GFR were moderately correlated with sGFR in both men and women. The overall staging accuracies of EPI-GFR and sGFR were significantly higher than that of cGFR. Among the patients staged, only those with GFRs in the range of 60–89 mL/min/1.73 m^2^ had moderate correlations between cGFR, EPI-GFR, and sGFR, while the remaining patients had low correlations.

**Conclusion:**

Researchers found that the equation developed for Chinese CKD patients performed well for patients with NB aged 20–25 years, while the race-neutral CKD-EPI equation performed better in NB patients aged 26–35 years.

## 1 Introduction

Neurogenic bladder (NB) is a dysfunction of the urinary bladder and urethra caused by diseases of the central and peripheral nervous systems (Liao, 2015a). The progression of this disease often leads to high pressure in the bladder, which in turn causes pathological changes such as vesicoureteral reflux, urethral fibrosis, and wall-segmental ureteral stenosis, leading to dilated upper urinary tract fluid retention and gradually impaired renal functions.

Patients with NB are at higher risk of developing chronic kidney disease (CKD) (Sung et al., 2018), which can lead to various complications and deterioration of renal function (Nseyo and Santiago-Lastra, 2017). The main causes of CKD are vesicoureteral reflux, recurrent urinary tract infections, hydronephrosis (HN), and kidney stones. Neurological lesions of the bladder caused by injuries or disorders related to bladder storage and voiding functions can exacerbate symptoms of NB. Patients with NB should receive blood urea nitrogen and serum creatinine (Cr) tests, electronic glomerular filtration rate assay (eGFR), renal ultrasonography to assess the renal structure, ^99m^Tc diethylenetriaminepentaacetic acid (DTPA) renal dynamic imaging to measure the unilateral renal GFR to assess renal structure and function, and cystoscopy and urodynamics to assess bladder structure and function, if necessary, at the time of follow-up (Panicker and Neurogenic, 2020). Liao (2015b) described a new grading system based on magnetic resonance urography for upper urinary tract dilatation (UUTD), including HN and ureteral dilatation (UD); this grading system allows objective and comprehensive assessment of HN and UD while emphasising and subdividing the renal parenchymal status, thereby assisting with the grading and longitudinal monitoring of UUTD, including its preoperative and postoperative observations (Liao et al., 2014). Unilateral renal GFR and serum Cr levels are also included in this grading system as important assessment criteria for upper urinary tract function, including renal function, in NB patients under follow-up.

The GFR is an important indicator of renal function and can be used to stage renal function, determine drug dosage, manage and determine the prognosis for NB-related adverse events, and mortality (Rabadi and Aston, 2016). Over the past two decades, automatic matching of clinical-laboratory-reported GFR values using Cr-based equations has been widely used in clinical practice. GFR equations provide a rapid method of estimating the GFR. The internationally recognised K/DOQI guidelines emphasise the importance of the GFR in the diagnosis of renal dysfunction, and the current guidelines recommend the use of Cr-based equations to calculate the GFR as an initial assessment (Miller et al., 2023).

A Cr-based GFR equation was developed for CKD patients in China in 2006 (Ma et al., 2006), and another new Cr-, age-, and gender-based CKD epidemiology collaboration (CKD-EPI) equation was developed in 2021 (Inker et al., 2021). The present study aims to use the GFR measured by dynamic renal scintigraphy (Gates method) as the standard for the application of two Cr-based GFR estimation equations in Chinese patients of different genders, age groups, and GFR stages who have been diagnosed with NB.

## 2 Materials and methods

### 2.1 Participants and data collection

We collected data from 99 NB patients from different regions of China who were outpatients and inpatients at the Department of Urology at the China Rehabilitation Research Centre from July 2016 to July 2023. We also diagnosed and classified the NB according to the NB diagnosis and treatment guidelines of the Chinese Society of Urology (CUA) of the Chinese Medical Association (CMA). Patients with acute kidney injury, severe oedema, pleural effusion or ascites, malnutrition, heart failure, or ketoacidosis were excluded from the study. This study was approved by the Ethics Committee of the China Rehabilitation Research Centre (approval number 2021–137-1). All participants provided written informed consent before data collection. The collected data included the age, gender, height, weight, body mass index (BMl), body surface area (BSA), neuropathy aetiology, as well as GFR values measured using ^99m^Tc-DTPA renal dynamic imaging (sGFR), the new serum creatinine (Cr)-based chronic kidney disease epidemiology collaborative group (CKD-EPI) equation (EPI-GFR), and the equation for the estimated GFR of CKD patients in China (cGFR). The fasting sera of the enrolled patients were also analysed for haemoglobin, Cr, urea nitrogen, and albumin at the laboratory of the Beijing Boai Hospital.

### 2.2 GFR measurement

^99m^Tc-DTPA renal dynamic imaging of the GFR measurement was obtained using the Intevo SPECT device from Siemens Medical Systems (Cleveland, OH, United States) (Gates, 1983). Twenty minutes prior to the examination, the patients were supplemented with 300–500 mL of water after breakfast. Prior to injection, a 6-s count was performed on a sample syringe containing 3 mCi of ^99m^Tc-DTPA. After injecting 111 MBq of ^99m^Tc-DTPA intravenously into the patient’s forearm, the patient was allowed to rest in the supine position for dynamic imaging. The kidney and bladder regions were then placed in the centre of the field of view of the gamma camera. The post-injection syringe counts were similar to the pre-injection counts, and the pre-count values were subtracted from the post-count values to obtain the total injected dose.

The region of interest (ROl) for each kidney was manually plotted on the frame within 1–3 min following injection; then, the background-corrected time–activity curves were generated, and 1-min unilateral renal uptake was calculated within 2–3 min after injection. After image acquisition, the patient’s height and visual acuity were obtained and entered into an on-line computer, and the GFR was calculated automatically according to the Gates algorithm and standardised to the BSA.

### 2.3 Measurement of serum Cr and calibration

The serum Cr measurements were performed in a dedicated laboratory affiliated to the Department of Laboratory Medicine at Beijing Boai Hospital. The sarcosine oxidase technique was used for the measurements along with the Mind BS2000-M automatic biochemical analyser (Shenzhen Mind Electronics Co., Ltd.), with a normal reference range of 0.49–1.30 mg/dL (44–115 µmol/L), as determined by the manufacturer. The coefficient of variation for both high and low chromium concentrations was less than 3%.

The cystatin C (CysC) value was not measured or used in the evaluations for the following reasons. (1) Cost and resource constraints: the detection of CysC requires specific reagents and equipment, and some hospitals may not be able to use this test because of cost and resource constraints. (2) Problems with standardisation of testing methods: in the past, there was no standard testing method for CysC, which affected its use in clinical practice. Although standardisation has improved in recent years along with improved comparability of results from different manufacturers, there may still be hospitals that have do not have access to updated testing methods.

### 2.4 Estimation of GFR from Cr-based equations

The serum Cr values were used to estimate the EPI-GFR (excluding ethnicity) and cGFR values (Table 1).

**TABLE 1:** Equations used to estimate the glomerular filtration rate (GFR).

GFR method	Sex	Cr	Equations
CKD-EPI equation	Female	Cr ≤ 0.7	142 ∗ (Cr/0.7)^−0.241^ ∗ (0.9938)^age^ ∗ 1.012
	Female	Cr > 0.7	142 ∗ (Cr/0.7) ^−1.200^ ∗ (0.9938)^age^ ∗ 1.012
	Male	Cr ≤ 0.9	142 ∗ (Cr/0.9)^−0.3.2^ ∗ (0.9938)^age^
	Male	Cr > 0.9	142 ∗ (Cr/0.9)^−1.200^ ∗ (0.9938)^age^
cGFR equation	Female	Cr	175 ∗ (Cr)^−1.234^ ∗ (Age)^−0.179^ ∗ 0.79
	Male	Cr	175 ∗ (Cr)^−1.234^ ∗ (Age)^−0.179^

Cr = creatinine: CKD-EPI equation = new Cr-based chronic kidney disease epidemiology collaboration (CKD-EPI) equation without race consideration; cGFR equation = estimated GFR equation for Chinese individuals, where Cr is in mg/L and age is in years.

### 2.5 Statistical methods

Quantitative data from the patients, including their age, height, weight, BMI, BSA, serum Cr, serum urea, serum albumin, equation-estimated GFR, and sGFR, are presented as mean ± standard deviation (SD) or median (25th and 75th percentiles). The absolute deviation is defined as the absolute value of the difference between the estimated GFR from the equations and sGFR. The deviation percentile is the ratio of this deviation to the sGFR expressed as a percentage. Similarly, the absolute deviation percentile is the absolute value of this percentage ratio. These parameters were used to evaluate the extent of deviation of the equation-derived GFRs compared to the sGFR. The median and interquartile range were used to evaluate the deviations. The 30% accuracy measure, denoted as P30, refers to the proportion of GFR values from each equation that fall within a 30% range of the sGFR value. This metric serves as an indicator of the precision of each equation used to estimate the sGFR. Bland–Altman plots were obtained to compare the 95% consistency bounds of the GFRs from each equation with the sGFR. Pearson correlation analysis was employed to evaluate the degree of correlation between the GFR values derived from each of the equations and the sGFR. Additionally, the Wilcoxon signed rank test was used to assess both the differences and absolute differences between the cGFR and EPI-GFR values. In this study, the results of SPSS software (version 27.0, SPSS, Inc., Chicago, IL, United States) and Medcalc for Windows version 11.0 (Medcalc Software, Mariekerke, Belgium) were considered to be significant at *p* < 0.05.

## 3 Results

Of the 99 patients with NB recruited in this study, 72 were men and 27 were women of age 30.6 ± 12.3 years, height 165 ± 5.5 cm, weight 63.5 ± 13.0 kg, and having BSAs of 1.9 ± 1.0 m^2^. The serum Cr value was 113.6 ± 81.4 µmol/L, and the sGFR was 79.8 ± 23.1 (normal range: 26.3–156.1) mL/min/1.73 m^2^. The mean cGFR and EPI-GFR values were 91.3 ± 4.4 (normal range: 8.5–217.2) mL/min/1.73 m^2^ and 86.9 ± 3.5 (normal range: 9.4–129.7) mL/min/1.73 m^2^, respectively (Table 2).

**TABLE 2:** Basic demographic information of the participants.

Characteristic (n = 99)	Mean ± SD (median) or n (%)
Male (n %)	72 (72%)
Female (n %)	27 (27%)
Age (years)	30.6 ± 12.3
Less than 20 years old	23 (23%)
20–25 years old	19 (20%)
26–35 years old	32 (32%)
36–65 years old	25 (25%)
BSA (m^2^)	1.95 ± 1.04
Plasma creatinine (µmol/L)	113.58 ± 81.39
sGFR (mL/min/1.73 m^2^)	79.8 ± 23.1
cGFR (mL/min/1.73 m^2^)	92.1 ± 42.4
EPI-GFR (mL/min/1.73 m^2^)	88.2 ± 33.9
GFR instalment	
15–29 (mL/min/1.73 m^2^)	1 (1%)
30–59 (mL/min/1.73 m^2^)	19 (19%)
60–89 (mL/min/1.73 m^2^)	45 (45%)
≥90 (mL/min/1.73 m^2^)	34 (34%)
Causes of NB	
Congenital or acquired urological diseases	10 (10%)
After pelvic surgery	11 (11%)
Diabetes	23 (23%)
Spinal cord injury	65 (66%)
Tethered cord syndrome	13 (20%)
Myelomeningocele	12 (18%)
Spinal cord tumour, haemangioma, lipoma, and teratoma	10 (15%)
Congenital spina bifida	30 (46%)
Trauma	20 (20%)
Lipogenic injury	1 (0.1%)
Other causes or causes unknown	18 (18%)

The results of comparison of the degrees of deviation of the equation-based GFRs relative to the sGFR for patients of different genders are shown in Table 3. The Wilcoxon signed rank sum test was performed for each equation-based GFR and sGFR, and the results suggest that all values are statistically significant (*p* < 0.05). The highest correlation between cGFR and sGFR was found in men (0.63), and the median EPI-GFR deviation and intercept were the least in men. In women, the EPI-GFR median absolute value of deviation (19.1) and median absolute value of deviation percentile (0.2) were the least, while the EPI-GFR 30% accuracy (88.8%) and total staging accuracy (92.3) were the highest.

**TABLE 3:** Analysis of deviation degrees in patients of different sexes.

Parameter	cGFR	EPI-GFR
Intercept (95% CI) (male)	−50.84 (−69.06 to 32.62)	−30.28 (−48.06 to −12.49)
Intercept (95% CI) (female)	−67.80 (−108.94 to 6.66)	−50.52 (−91.64 to −9.39)
Slope (95% CI) (male)	0.74 (0.54–0.94)	0.46 (0.26–0.67)
Slope (95% CI) (female)	0.87 (0.42–1.31)	0.63 (0.16–1.09)
Median of difference (mL/min/1.73 m^2^) (25%, 75% percentile) (male)	8.7 (−16.6, 36.7)	3.0 (−13.2, 32.0)
Median of difference (mL/min/1.73 m^2^) (25%, 75% percentile) (female)	12.2 (−2.1, 29.7)	7.9 (−6.4, 21.7)
Absolute median difference (mL/min/1.73 m^2^) (25%, 75% percentile) (male)	25.0 (13.4, 37.7)	20.7 (11.9, 34.5)
Absolute median difference (mL/min/1.73 m^2^) (25%, 75% percentile) (female)	27.3 (7.7, 46.3)	19.7 (7.4, 33.1)
Median percentile difference (mL/min/1.73 m^2^) (25%, 75% percentile) (male)	0.1 (−0.24, 0.44)	0.1 (−0.2, 0.4)
Median percentile difference (mL/min/1.73 m^2^) (25%, 75% percentile) (female)	0.2 (0.0, 0.4)	0.1 (−0.1, 0.2)
Median absolute difference percentile (mL/min/1.73 m^2^) (25%, 75% percentile) (male)	0.3 (0.2, 0.5)	0.3 (0.1, 0.5)
Median absolute difference percentile (mL/min/1.73 m^2^) (25%, 75% percentile) (female)	0.3 (0.2, 0.5)	0.2 (0.1, 0.4)
Correlation coefficient (r) (male)	0.63	0.56
Correlation coefficient (r) (female)	0.47	0.52
Accuracy		
±30% (male)	22.2%	22.2%
±30% (female)	38.5%	88.8%
Total staging (male)	66.7%	69.7%
Total staging (female)	46.2%	92.3%
Wilcoxon signed rank test (P) (male)	<0.001	<0.001
Wilcoxon signed rank test (P) (female)	<0.001	<0.001

cGFR = GFR estimated using the GFR equation for Chinese individuals; EPI-GFR = GFR estimated using the CKD-EPI equation without race consideration; sGFR = GFR measured by ^99m^TC-DTPA renal dynamic imaging.

The results of comparison of the degrees of deviation of the equation-based GFRs relative to the sGFR for patients of different ages are shown in Table 4. The Wilcoxon signed rank sum test results between the equation-based GFRs and sGFR indicate statistical significance (*p* < 0.05). In patients aged 20–25 years, cGFR was highly correlated with sGFR (0.78), intercept of linear regression of cGFR was much lower (0.59), median EPI-GFR deviation percentile and median absolute value of the deviation percentile were the least, and 30% accuracy was the highest (54.55%); however, the total cGFR staging accuracy was found to be the highest (95.94%). In patients aged 26–35 years, EPI-GFR had the highest correlation with sGFR (0.74) and the least absolute EPI-GFR deviation (16.5).

**TABLE 4:** Analysis of deviation degrees in patients of different age groups.

Parameter	cGFR	EPI-GFR
Intercept (95% CI) (less than 20 years old)	0.33 (2.46–4.21)	−0.11 (−0.48 to 0.26)
Intercept (95% CI) (20–25 years old)	0.59 (0.20–0.99)	0.77 (0.33–1.22)
Intercept (95% CI) (26–35 years old)	0.42 (0.07–0.77)	0.60 (0.25–0.95)
Intercept (95% CI) (36–65 years old)	0.47 (−0.19 to 1.13)	0.97 (0.26–1.68)
Slope (95% CI) (less than 20 years old)	−0.02 (−0.03 to 0.01)	0.01 (0.00–0.01)
Slope (95% CI) (20–25 years old)	0.01 (0.00–0.01)	0.00 (0.00–0.01)
Slope (95% CI) (26–35 years old)	0.01 (0.00–0.01)	0.01 (0.00–0.01)
Slope (95% CI) (36–65 years old)	0.01 (0.00–0.02)	0.00 (0.00–0.01)
Median of difference (mL/min/1.73 m^2^) (25%, 75% percentile) (less than 20 years old)	−16.4 (−28.0, 33.9)	−12.0 (−26.8, 34.9)
Median of difference (mL/min/1.73 m^2^) (25%, 75% percentile) (20–25 years old)	−8.0 (−24.9, 26.7)	−6.1 (−22.3, 23.1)
Median of difference (mL/min/1.73 m^2^) (25%, 75% percentile) (26–35 years old)	7.4 (−10.2, 27.7)	7.7 (−6.4, 21.2)
Median of difference (mL/min/1.73 m^2^) (25%, 75% percentile) (36–65 years old)	24.6 (9.3, 43.5)	18.0 (4.4, 36.5)
Absolute median difference (mL/min/1.73 m^2^) (25%, 75% percentile) (less than 20 years old)	29.4 (16.5, 57.8)	33.3 (13.9, 50.2)
Absolute median difference (mL/min/1.73 m^2^) (25%, 75% percentile) (20–25 years old)	24.9 (9.5, 37.4)	22.3 (6.2, 33.1)
Absolute median difference (mL/min/1.73 m^2^) (25%, 75% percentile) (26–35 years old)	19.5 (8.0, 32.6)	16.5 (7.1, 26.5)
Absolute median difference (mL/min/1.73 m^2^) (25%, 75% percentile) (35–65 years old)	25.7 (14.5, 43.5)	22.4 (12.8, 36.5)
Median percentile difference (mL/min/1.73 m^2^) (25%, 75% percentile) (less than 20 years old)	−0.2 (−0.5, 0.4)	−0.1 (−0.4, 0.4)
Median percentile difference (mL/min/1.73 m^2^) (25%, 75% percentile) (20–25 years old)	−0.1 (−0.3, 0.4)	0.0 (−0.3, 0.3)
Median percentile difference (mL/min/1.73 m^2^) (25%, 75% percentile) (26–35 years old)	0.1 (−0.1, 0.3)	0.1 (−0.1, 0.2)
Median percentile difference (mL/min/1.73 m^2^) (25%, 75% percentile) (36–65 years old)	0.3 (0.2, 0.7)	0.3 (0.1, 0.5)
Median absolute difference percentile (mL/min/1.73 m^2^) (25%, 75% percentile) (less than 20 years old)	0.5 (0.2, 0.7)	0.4 (0.2, 0.6)
Median absolute difference percentile (mL/min/1.73 m^2^) (20–25 years old)	0.4 (0.1, 0.4)	0.3 (0.0, 0.4)
Median absolute difference percentile (mL/min/1.73 m^2^) (26–35 years old)	0.2 (0.1, 0.4)	0.2 (0.1, 0.3)
Median absolute difference percentile (mL/min/1.73 m^2^) (36–65 years old)	0.4 (0.2, 0.7)	0.3 (0.2, 0.5)
Correlation coefficient (r) (less than 20 years old)	0.35	0.57
Correlation coefficient (r) (20–25 years old)	0.78	0.75
Correlation coefficient (r) (26–35 years old)	0.70	0.74
Correlation coefficient (r) (36–65 years old)	0.47	0.49
Accuracy		
±30% (less than 20 years old)	16.67%	16.67%
±30% (20–25 years old)	27.27%	31.82%
±30% (26–35 years old)	54.55%	54.55%
±30% (36–65 years old)	29.17%	41.67%
Total staging (less than 20 years old)	38.89%	44.44%
Total staging (20–25 years old)	95.94%	93.22%
Total staging (26–35 years old)	77.27%	90.91%
Total staging (36–65 years old)	42.31%	54.17%
Wilcoxon signed rank test (P) (less than 20 years old)	<0.001	<0.001
Wilcoxon signed rank test (P) (20–25 years old)	<0.001	<0.001
Wilcoxon signed rank test (P) (20–25 years old)	<0.001	<0.001
Wilcoxon signed rank test (P) (36–65 years old)	<0.001	<0.001

cGFR = GFR estimated using the GFR equation for Chinese individuals; EPI-GFR = GFR estimated using the CKD-EPI equation without race consideration; sGFR = GFR measured by ^99m^TC-DTPA renal dynamic imaging.

The results of comparison of the degrees of deviation of each equation-based GFR from the sGFR in patients with different GFR stages are shown in Table 5. The Wilcoxon signed rank sum test results were compared for the equation-based GFRs and sGFR and found to be statistically significant in the staging range of 60 ≦ sGFR ≤ 89 mL/min/173 m^2^ (*p* < 0.05); here, the cGFR was weakly correlated with sGFR (0.35), and the EPI-GFR was also weakly correlated with sGFR (0.42).

**TABLE 5:** Analysis of deviation degrees in patients with different GFR stages.

Parameter	cGFR	EPI-GFR
Correlation coefficient (r) (30 ≤ sGFR ≤ 59 mL/min/1.73 m^2^)	0.13	0.15
Correlation coefficient (r) (60 ≤ sGFR ≤ 89 mL/min/1.73 m^2^)	0.35	0.42
Correlation coefficient (r) (90 ≥ sGFR mL/min/1.73 m^2^)	0.18	0.22
Wilcoxon signed rank test (P) (30 ≤ sGFR ≤ 59 mL/min/1.73 m^2^)	0.904	0.778
Wilcoxon signed rank test (P) (60≤ sGFR ≤ 89 mL/min/1.73 m^2^)	0.018	0.016
Wilcoxon signed rank test (P) (90 ≥ sGFR mL/min/1.73 m^2^)	0.118	0.347

## 4 Discussion

The application of GFR equations based on the serum Cr levels in Chinese patients with NB is extremely rare. The results of this study show that the cGFR was appropriate for NB patients aged 20–25 years, whereas the race-neutral EPI-GFR was suitable for NB patients aged 26–35 years. Thus, we encourage clinicians to pay close attention to these evaluation measures so as to quickly and more accurately assess the early renal functions of such patients and improve their prognosis thereof.

**FIGURE 1:**
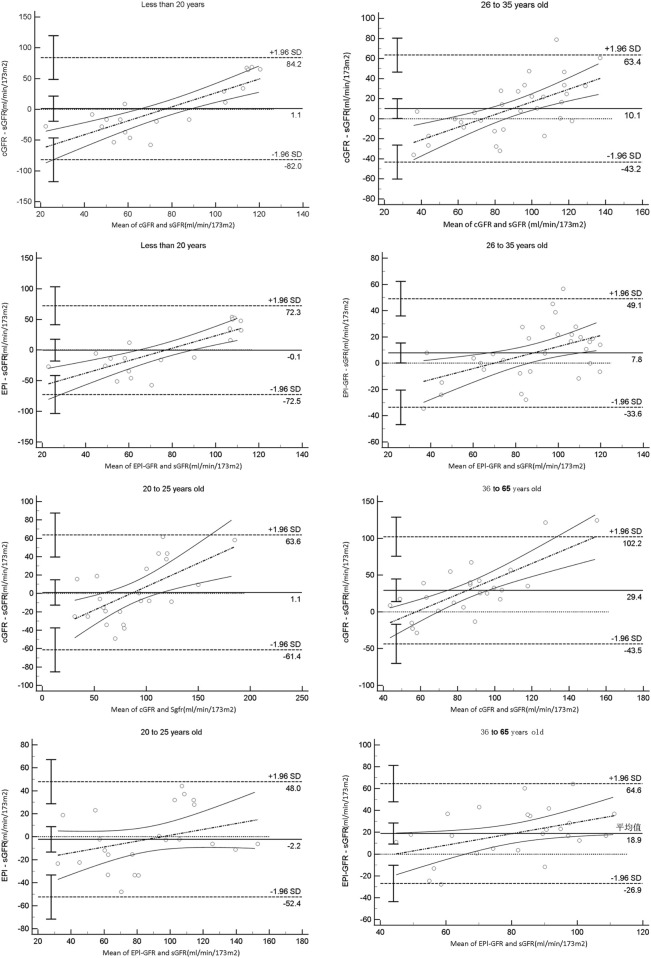
Bland–Altman plot analysis of the 95% consistency limits in patients of different age groups.

In this study, we found that the equation-based GFRs were highly correlated with the sGFR values in patients aged 20–35 years and that the intercept of the linear regression of the cGFR was much lower in patients aged 20–25 years. The difference between EPI-GFR and sGFR was similar to that between cGFR and sGFR, but the absolute deviation between EPI-GFR and sGFR was significantly lesser than that between cGFR and sGFR in patients aged 26–35 years. The total staging accuracy with cGFR was better in patients aged 20–25 years, whereas the total staging accuracy with EPI-GFR was better in patients aged 26–35 years. In patients younger than 26 years, the median deviation between cGFR and EPI-GFR was less than zero, suggesting underestimation of the patient’s GFR; in patients older than 25 years, the median deviation between the cGFR and EPI-GFR was greater than 0, suggesting overestimation of the patient’s GFR. From Figure 1, the Bland–Altman plots show consistent differences in the equation-based GFRs versus sGFR values, with the narrowest 95% confidence intervals being observed for the differences between EPI-GFR and sGFR in NB patients aged 20–25 years (22.15 mL/min/1.73 m^2^) as well as 26–35 years (15.52 mL/min/1.73 m^2^). In summary, the Cr-based cGFR showed good applicability in NB patients aged 20–25 years, whereas the race-neutral EPI-GFR had better applicability in NB patients aged 26–35 years.

Evaluation of the direct effect of the serum Cr level on the GFR equation (Alareqi et al., 2022) and the serum Cr concentration itself are susceptible to human muscle mass, age, gender, diet, exercise, nutritional status, chronic diseases, and other factors (Blaufox et al., 1996). Renal tubules and collecting ducts secrete small amounts of Cr, so the Cockcroft–Gault equation systematically overestimates the GFR; this is more pronounced in oedematous and obese patients, especially those with type 2 diabetes associated with obesity (Chudleigh et al., 2008; Fabbian et al., 2013). CysC-based equations have been found to be the most accurate method of assessing GFRs (Delanaye and Mariat, 2013; Cha et al., 2010). Recently, several studies have shown that the currently used GFR equations combining Cr and CysC are more accurate than those using only Cr or CysC with respect to the adult CKD population (Grubb et al., 2015) and renal transplant donors (Ma et al., 2007); however, this approach has not yet been evaluated for use in NB patients. Inulin renal clearance is the gold standard for GFR measurement but is known to be invasive and costly (Grubb et al., 2015); in addition, it requires precise intravenous infusion of inulin to achieve steady-state plasma concentration and frequent timed urine collection to completely empty the bladder. However, it is also well known that the vast majority of NB patients are unable to empty their bladders well, making it impractical to measure inulin clearance in patients with NB. The two linear regression formulas considered here are based solely on the characteristics of the distribution of CKD patients; the coefficients given in these formulas are only applicable to some CKD patients and may not be applicable to the distribution of some NB patient groups.

Despite the encouraging findings of this study, there are some notable limitations. First, the number of patient cases considered was small. Second, the distribution of cases with different GFR stages was not uniform in this study. Third, our previous study showed that sGFR itself is somewhat defective in ^99m^Tc-DTPA renal dynamic imaging and that the sGFR in Chinese CKD patients varied widely with low precision and accuracy (Ma et al., 2007). The Gates method is acceptable for assessing the GFRs in patients with normal to moderately reduced renal functions but is less accurate in patients with severe renal insufficiency. This means that the ^99m^Tc-DTPA renal dynamic imaging method may not sufficiently provide an accurate GFR assessment in patients with severely impaired renal function. Therefore, it may not be suitable as a reference value for GFR based on renal dynamic imaging. However, our study also has the merit that the Cr-based GFR equation is currently widely advocated and promoted by clinicians.

## 5 Conclusion

The present study shows that in Chinese NB patients with gender and GFR staging, Cr-based GFR equations (including the race-neutral CKD-EPI and Chinese GFR estimation equations) show unsatisfactory performances, as also reported by Ma et al. (2007). However, the results were different for patients aged 20–25 years. The cGFR was found to be highly correlated with sGFR with high overall staging accuracy, which can be used as the preferred method for early renal function assessment in NB patients. In patients aged 26–35 years, the EPI-GFR showed high correlation with sGFR along with good bias and total staging accuracy, which can be used as the first-choice approach for early renal function assessment in NB patients.
